# Technologies for the point-of-care diagnosis of malaria: a scoping review

**DOI:** 10.1186/s40249-025-01329-1

**Published:** 2025-06-23

**Authors:** Florinda Coro, Carmelo De Maria, Valentina D. Mangano, Arti Ahluwalia

**Affiliations:** 1 Department of Information Engineering and Research Centre ‘E. Piaggio’, University of Pisa, Pisa, Italy; 2 Ubora Association, Pisa, Italy; 3 Department of Translational Research and New Technologies in Medicine and Surgery, University of Pisa, Pisa, Italy

**Keywords:** Malaria, Diagnosis, Point-of-care, ASSURED, Scoping review

## Abstract

**Background:**

Malaria continues to pose a significant health challenge, particularly in low-resource settings (LRS), where access to reliable and timely diagnostics is often limited. In this context, point-of-care (POC) in vitro diagnostics (IVDs) play a key role in supporting early detection and treatment. The aim of this scoping review was to better understand the landscape of malaria IVD technologies, with the aim of identifying both their strengths and limitations to guide and accelerate the development of POC diagnostics suitable for endemic regions and LRS. To support this analysis, the ASSURED (Affordability, Sensitivity, Specificity, User-friendliness, Rapidity, Equipment-free, Deliverability) criteria were applied to rank each technology in terms of its potential for POC applications in LRS.

**Methods:**

A literature search was conducted in PubMed and Web of Science for original research articles on malaria POC diagnostic devices published in English over the last 20 years (2003–2023). Records were screened based on eligibility criteria. For each paper, we identified biomarkers, biological specimens used, analytical methods, and readout technologies. Each record was ranked from low to high for its compatibility with the seven ASSURED criteria and for the Technology Readiness Level.

**Results:**

The final dataset included 118 records. Of the methods considered, immunoassays were the most frequently reported (41.5%), followed by loop-mediated isothermal amplification (LAMP, 22.8%), polymerase chain reaction (PCR, 6.7%) and optical microscopy (4.2%). The limit of detection was highest for LAMP and PCR. Biomarkers employed for diagnosis included the *Plasmodium* parasite, parasite protein antigens and hemozoin. Blood was the most commonly employed biological specimen (76.2%), followed by urine and saliva (5.1%). Despite a focus on malaria IVDs for POC applications, only 8% of the records mentioned ASSURED criteria, with most studies manifesting low compatibility with the criteria.

**Conclusions:**

Although meeting the ASSURED criteria remains challenging, microscopy is still the gold standard because of its diagnostic accuracy. Recent developments in low-cost, high-magnification lenses and innovative manufacturing techniques have enabled the production of microscopy devices in LRS. Combined with advancements in image processing and shape recognition through machine learning, there is strong potential for intellectual and economic investments to enhance microscopy for POC malaria diagnostics.

**Graphic abstract:**

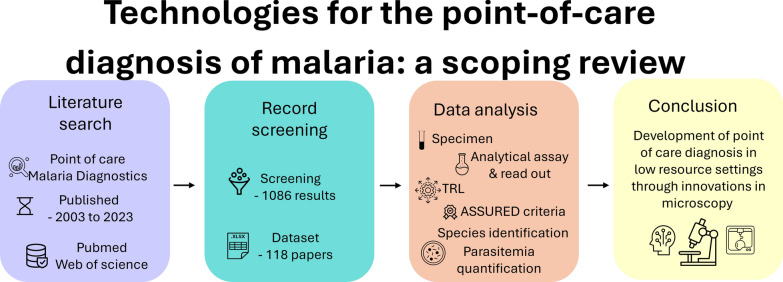

**Supplementary Information:**

The online version contains supplementary material available at 10.1186/s40249-025-01329-1.

## Background

Malaria is a parasitic disease caused by Apicomplexan protozoans of the genus *Plasmodium* and transmitted by female *Anopheles* mosquitoes [1]-[2]. Most infections in humans are caused by five species namely: *P. falciparum, P. vivax, P. ovale, P. malariae*, and *P. knowlesi* [3]-[4]. When an infected mosquito bites a human host for a blood meal, sporozoites are injected into the skin and about 40% of them enter the blood vessels. Within 30–60 min, sporozoites are transported to the liver where they invade hepatocytes and undergo asexual replication by schizogony. After about 2–15 days, depending on the species, merozoites are released into the circulation where they invade and replicatein red blood cells with multiple cycles that are the cause of clinical signs and symptoms. Upon invasion of RBC some merozoites undergo sexual differentiation as opposed to schizogony, and develop into gametocytes. When a mosquito bites an infected individual, it ingests gametocites that undergo maturation and fertilization producing an oocyst and thereby leading to the generation of sporozoites s able to start a new cycle of infection.

Although it is preventable and treatable, malaria remains a life-threatening disease and a major threat to global public health, with ongoing transmission in 85 countries [5]. The World Health Organization (WHO) estimated that there were 249 million clinical cases of malaria and 619,000 related deaths in 2022. Of the malaria cases, 95% occurred in sub-Saharan Africa, and 80% were in children under 5 years old [6]. Reducing malaria morbidity and mortality by 90%, preventing the re-establishment of the disease in malaria-free countries, and decreasing the number of endemic countries is the objectives of target 3.4 of the 2030 United Nations (UN) Agenda for sustainable development [6]. The key factor for achieving these goals is increased coverage of preventive, diagnostic and treatment services.

Regarding diagnosis, the WHO strongly recommends parasitological confirmation of suspected clinical cases prior to administering anti-malarial treatment, to reduce the unnecessary use of drugs that contribute to the development of resistance [7], prevent misdiagnosis and thereby ensure appropriate and effective treatment. Microscopy is considered the gold standard for malaria diagnosis because it allows parasite detection, species identification and determination of parasite density, providing definite evidence of infection and important information for accurate treatment [8]. However, its implementation is challenging in low resource settings (LRS), as laboratory equipment, electricity and expert microscopists are needed [9].

Point-of-care (POC) in vitro diagnostic (IVD) tools constitute a valuable opportunity to make malaria diagnosis widely available even in remote and rural areas in LRS—areas which might have difficulties in allocating financial resources, maintaining supply chains for medical devices, and coping with extreme environmental conditions. POC technologies have garnered significant interest since their introduction on the market as they can provide a fast, accurate, affordable, and easy to use method for detecting infections [10].

In 2006, The WHO defined the “ASSURED” benchmark criteria for the development of POC IVDs for use in LRS in low- and middle-income countries. The acronym stands for *Affordable, Sensitive, Specific, User-friendly, Rapid and robust, Equipment-free, and Deliverable to the end user* [11]. *Affordability* refers to the price of a single test for the end user. Estimating how much a product must cost is critical; according to the Gross National Income (GNI) thresholds established by the World Bank, it is reasonable to assume that the cost should be between USD 0.50 and USD 1 for a single test [12]. Given the technological focus of this scoping review, *affordability* was scored via Technology Readiness Level (TRL), a well-established indicator referring to the maturity of a technology, based on the rationale that the lower the technological maturity, the more unscaled the manufacture or production is, and therefore the higher the price [13].

*Sensitivity* and *specificity* refer to the diagnostic accuracy and therefore to the capacity of minimizing false negatives and false positives, respectively. Accuracy of diagnosis was assessed using the limit of detection (LOD) of each methodology, as well as its ability to identify parasites species and quantify parasite density. *User-friendliness* is associated with the training necessary to use the IVD with minimal human error, a crucial factor, especially in LRS because of the reduced number of specialised laboratory personnel [14]. *Rapidity* is defined as a reduced waiting time between sample collection and diagnostic results; it is fundamental to improving patient outcomes, especially in LRS where accessing health services might require long and arduous travel [15]. *Robustness* is related to the capacity of a test to resist environmental stresses and maintain a standard quality over time. *Equipment-free* is defined as the possibility to perform the IVD test without the need for a laboratory environment or specialized instruments. *Deliverable to end-user* refers to the ease of procurement and storage of the IVD at the POC, including the need for refrigeration for thermal stability of the reagents. In 2019, Land and colleagues [16] proposed an updated version of the ASSURED criteria, called REASSURED, adding *real-time connectivity* and *ease of specimen collection environmental friendliness* to the first seven criteria. Since the criteria were proposed only in 2019, most of the records included in this study could not take them into account. For this reason, we based our technology assessment on the original ASSURED criteria [15]. Given the low accessibility of microscopy for malaria diagnostics in endemic areas, POC alternatives have been widely explored. Among these, rapid diagnostic tests (RDTs) have been on the market since the 2000s and have been recommended by the WHO when microscopy is not available for malaria diagnosis. RDTs detect parasite antigens in peripheral blood. They do not require any laboratory equipment and the response time is rather rapid (15–30 min). Most RDTs are based on the detection of *Plasmodium* lactate dehydrogenase (pLDH) or *P. falciparum* Histidine-Rich Protein II (HRP2). The major limitation of RDTs is false negative results due to their suboptimal sensitivity at low parasite densities and with non-falciparum infections [17]. On the other hand, molecular diagnostic methods based on polymerase chain reaction (PCR) are very sensitive and specific. However, they take considerable time (3–5 h) for diagnosis and require skilled personnel and appropriate facilities with sophisticated laboratory equipment, running water and electricity [18]. Thus, despite their capacity to detect very low levels of parasitaemia (1–5 parasites/µl or even lower), PCR-based analyses are not easily deployable in LRS [19]. Loop-mediated isothermal amplification (LAMP) is a promising POC molecular method since it has a similar sensitivity to PCR but requires a much shorter time for diagnosis (45–60 min), and simpler but less expensive equipment [20].

As malaria is endemic mainly in areas where specialised laboratories are sparse, effective POC IVDs are urgently needed [21]. To date, a few scoping reviews have been published on malaria prevention and control strategies [22], or case management [23]. However, to our knowledge no such reviews have been conducted on malaria diagnostic tools. To address this gap, we performed a critical comparison of malaria POC IVDs described in scientific articles published in the last 20 years. The aim was to identify current limitations to the effective implementation in LRS that should be addressed by future research and innovation efforts.

## Methods

### Search strategy

Given the large coverage of literature on malaria diagnosis, a scoping review was used to describe the scientific publications on malaria POC IVDs and identify technological limitations which new research should address [24]. The search strategy was based on following research questions:

- What is the state of art in the development of POC IVDs for the diagnosis of malaria?
- How many scientific articles on the topic were published from 2003 to 2023?
- What are the specimens, analytes, analytical methods and readout technologies involved?
- Are IVDs adequate for use in LRS according to ASSURED criteria?
- What is the state of development of the IVDs as measured by the Technology Readiness Level (TRL)?
- Are there any technical limitations which new research and innovation need to or could address?
- Were articles authored by scientists working in malaria endemic areas?

The study was conducted in accordance with the JBI Manual for evidence synthesis (April 2021) and is congruent with the PRISMA ScR Checklist [25].

**Fig. 1:**
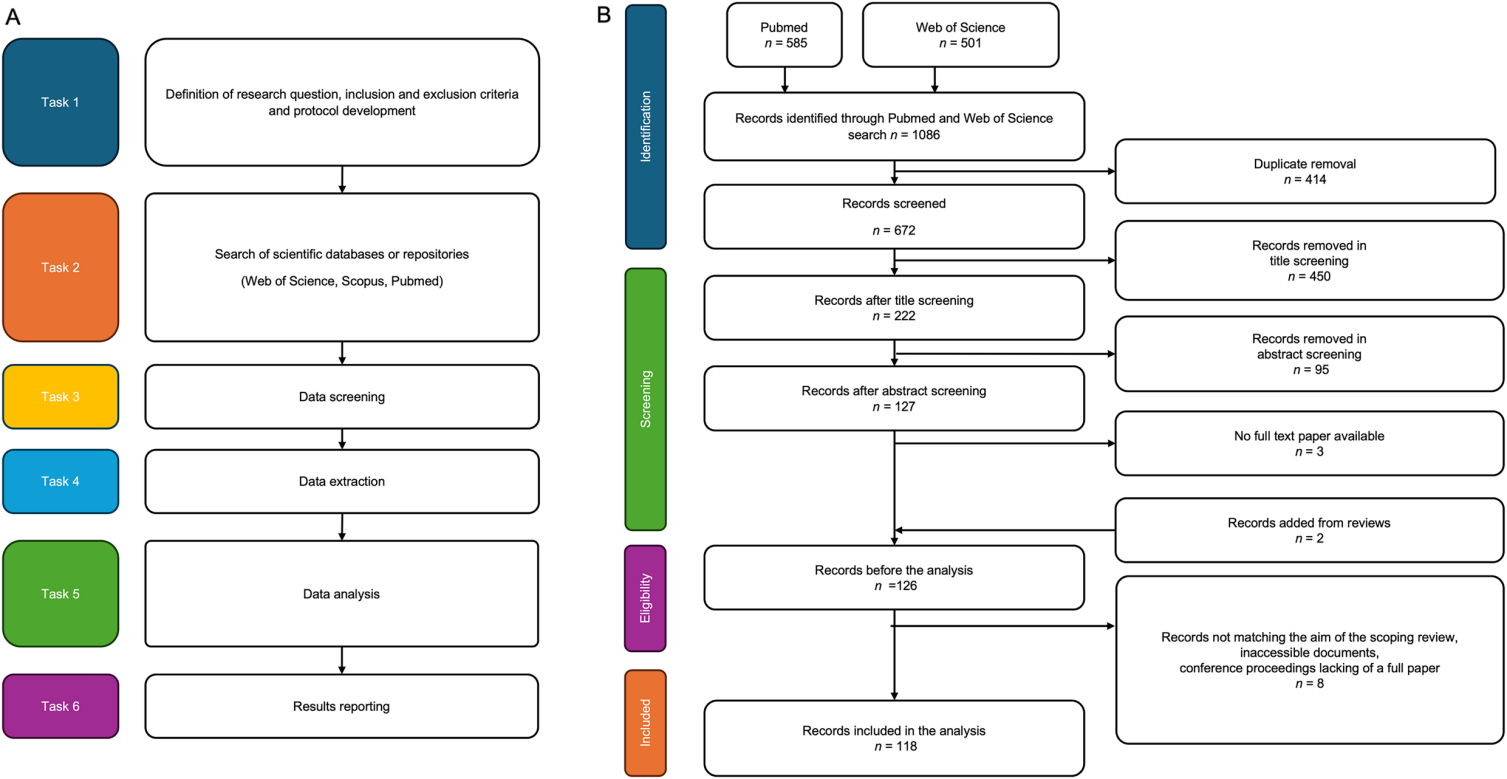
**A** Overview of the search strategy. **B** Flow diagram of the screening protocol according to PRISMA guidelines

After formulating the questions, the search strategy, outlined in Fig. 1A was implemented.

A preliminary search was conducted in Scopus, PubMed and Web of Science databases to identify the terms needed to extract relevant records from the literature. This preliminary search also showed that Scopus did not allow the distinction between keywords entered by the authors or automatically assigned, resulting in a very large dataset including many records that were irrelevant to the research questions. Scopus was therefore not included in the search. The terms used in the search were: Malaria/*Plasmodium*, Diagnosis, Point-of-care, combined and varied with Boolean operators and wildcards as reported in the Supplementary Information (SI, Table SI2). The final search was conducted in PubMed and Web of Science databases.

### Inclusion and exclusion criteria

The records were selected according to the inclusion and exclusion criteria presented in the Supplementary informations(SI) in Table S1. Original research articles with a focus on malaria POC IVDs were included. Review papers (as a primary source of information), case studies, editorials, commentaries, opinion papers, letters and grey literature were excluded. The time frame of the research was limited to the last 20 years since this time window was deemed sufficient to detect the presence of any technological limitations. Although we recognise that it may introduce a potential bias, only records written in English were included, since all the authors are proficient in English, and it is the predominant language of scientific publishing.

Each record was screened according to the eligibility criteria by two coauthors. In cases of disagreement between coauthors, a third coauthor’s opinion solved conflicts. Reviews were not included in the study but were used to inspect the references for relevant records to be added in the final dataset.

### Evidence extraction and analysis

For each record included in our study, we identified the biological specimen used in testing/validation, the biomarker (i.e. analyte) to be detected, the analytical method (i.e. assay) employed, and the readout (i.e. signal transduction) technology.

The assays were based on immunoassays, LAMP, PCR, optical microscopy, aptamer binding, Clustered Regularly Interspaced Short Palindromic Repeat (CRISPR), magneto-optical assay, probe hybridization, rolling-Circle-Enhance-Enzyme-Activity-Detection, fluorescence resonance energy transfer, precipitation polymerization assay, ATR-FTIR spectroscopy, optical absorption, near infra-red spectrophotometry, and refractive index-based methodologies. Assays described in less than 3 papers were grouped as “other” when generating figures. Each record was then examined in relation to the ASSURED criteria [12]. For each criterion, the compatibility of the IVD reported in the record with POC applications was ranked from 1 (low) to 3 (high) as described in Table 1.

**Table 1:** Characteristics employed to rank each POC technology according to ASSURED criteria

	Low (1)	Medium (2)	High (3)
Affordability	1 $\le TRL \le$ 4	5 $\le TRL \le$ 7	TRL 8–9
Sensitivity	< 95%	> 95% and < 98%	> 98%
Specificity	< 95%	> 95% and < 98%	> 98%
Usability	Requires long training	Requires short training	Minimal training
Rapidity and robustness	More than 2 h to obtain result	Between 30 min and 2 h to obtain result	Up to 30 min to obtain result
Equipment free	Requires complex laboratory instruments	Requires basic laboratory instruments	Does not require laboratory instruments
Deliverable to end user	Need cooling or refrigeration for shipment	–	Does not need cooling or refrigeration for shipment

*TRL* technology readiness level

As far as *affordability* is concerned, as summarised by Hutchings [26], for any healthcare technology, the most important factor is the clinical benefit for the patient followed by other users’ benefits, healthcare costs, and social gain for patients. Prices are also influenced by intellectual property, prices of competitive products, rarity of the disease/medical condition and unmet clinical needs [26, 27]. The manufacturing process—which includes investments, cost of materials and technological advancements for production as well as running costs—also has a strong impact, as does the cost of reagents. Due to the technical focus and nature of the records reviewed, it was not possible to conduct a comprehensive *affordability* assessment based on Hutching’s framework. Instead, we adopted the TRL: low *affordability* was considered for TRL below 4 (immature technology), medium *affordability* for TLR 4–7 (mature technology), and high *affordability* for TLR 8–9 (complete market-ready products). The procedure for assessing the TRL is described later in this section.

*Sensitivity* refers to the proportion of false positive results of a novel IVD compared to a reference IVD, while *specificity* to the proportion of false negative results. These parameters define the diagnostic accuracy of a novel IVD, but they both depend on the reference IVD employed by authors and are thereby not comparable between different records. For this reason, to assess the diagnostic accuracy of IVDs we considered the limit of detection (LOD; i.e. the minimum detectable parasite density) of the IVD as an indicator of analytical sensitivity [28], and the ability to identify the *Plasmodium* species and to determine the parasite density as indicators of specificity.

Briefly, an LOD value > 200 parasites/µl (was categorized as low accuracy (1), those between 5 and 200 parasites/µl as medium accuracy (2), and those < 5 parasites/µl as high accuracy (3). The inability to identify *Plasmodium* species (SID) was classified as indicating low accuracy (1), the ability to distinguish between 1 or 2 species as medium accuracy (2), and the ability to distinguish more than 2 species as high accuracy (3). The inability to quantify parasite density (QPD) was considered low accuracy (1), while the ability to quantify it was considered high accuracy (3).

In cases where a specific ASSURED criterion was not cited in the publication, a rating was assigned based on the available information. In cases of insufficient information, the criterion evaluation was considered “not applicable” and not included in the ranking.

After records were ranked for each ASSURED criterion, rankings were averaged across records per analytical method, to estimate the overall assay compatibility of a given IVD technology with POC applications: low (1), low/medium (1.5), medium (2), medium/high (2.5), high (3).

Given the importance of pushing technological transitions in the battle against malaria, we also used TRL as an indicator of technological maturity. In this case, TRL was based on the sample size described in each record, as this reflects the stage of development of the IVD, its scalability, reliability and readiness for operational use and therefore provides a reasonable indication of its potential for commercial expansion. TRL 1–2 was assigned to records reporting only the validation of the concept using up to 10 samples. TRL 3–4 was for records with laboratory validation (10–50 samples). TRL 5–6 was for records showing in-field validation (50–100 samples), TRL 7–8 was for records on final products not yet commercialised (> 100 samples), and TRL 9 for commercial products. Further details on data extraction and storage methods are provided in the SI.

## Results

The dataset resulting from the search contained 1086 records, which were screened according to the framework depicted in Figure 1B. After screening, 118 records were included for analysis.

### Biomarkers, assays and biological samples used for malaria diagnosis

**Fig. 2:**
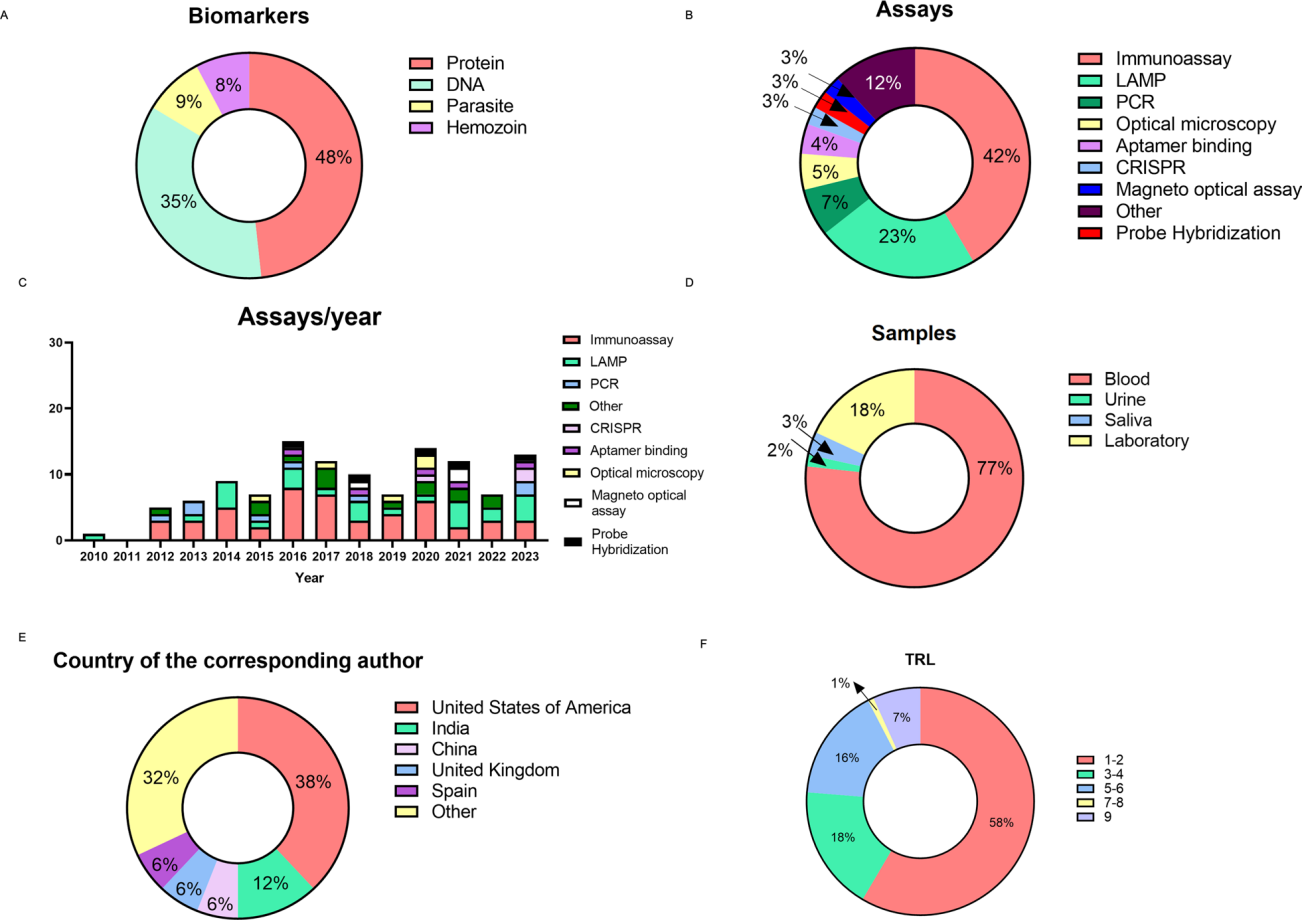
Reports included in the study grouped according to: **A** Biomarker; **B** Analytical method (assay); **C**; Year; **D** Biological specimen. **E** Corresponding author countries; **F** TRL. Abbreviations, *LAMP* loop-mediated isothermal amplification *PCR* polymerase chain reaction, *CRISPR* Clustered Regularly Interspaced Short Palindromic Repeats

*Plasmodium* proteins (47.5%) and DNA (34.9%) are the most common biomarkers employed for diagnosis. Detection of the parasite and hemozoin are far less frequent (< 10%) (Fig. 2A). Figure 2B illustrates the number of publications grouped according to the analytical method. Immunoassays are the most frequently reported method, accounting for over 41.5% of the total, followed by LAMP (22.8%) and PCR (6.7%). Optical microscopy is described in only 4.2% of the records. Although the search was conducted in the period 2003–2023, no records were published before 2010 (Fig. 2C). Over ¾ of the papers use blood to test the diagnostic systems. Laboratory samples—such as malaria cell cultures or media spiked with antigens—are used in 18% of cases. Very few studies use other biological fluids such as saliva or urine (Fig. 2D).

### Reporting of ASSURED-relevant criteria

Interestingly, even though our study was focused on malaria IVDs for POC applications, only 8% of the records mentioned the ASSURED criteria. Only 28% of them have at least one author affiliated to an institution located in malaria endemic countries. Of these, 56.4% authors are from South-East Asia, 35.8% from sub-Saharan Africa and 7.6% from South America. As far as corresponding authors are concerned, most are from the USA, followed by India, China, Spain and the United Kingdom (Fig. 2E). Most (58.4%) of the papers report emergent technology at a TRL of 1–2 while only 7.8% cover TRL of 7–9 (Fig. 2F). Table SI3 in the SI specifies all the analytical methods, the read-out technologies identified for each biomarker, and the associated number of papers.

**Fig. 3:**
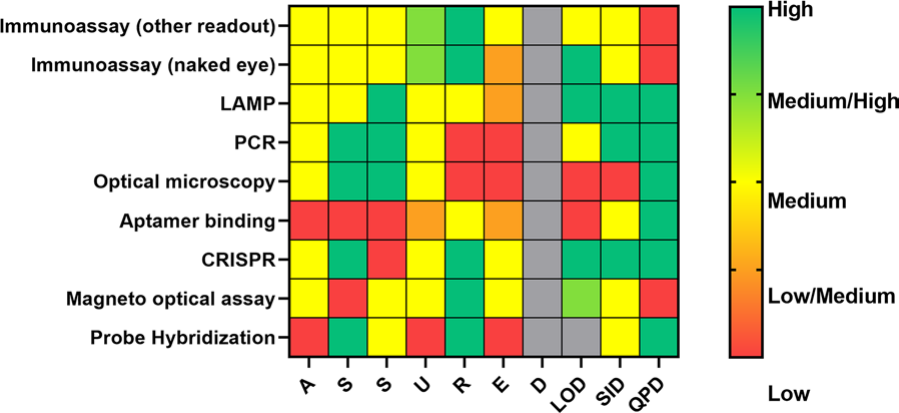
Heatmap indicating the compatibility of analytical assays included in the study with ASSURED criteria, limit of detection (LOD), species identification (SID) and parasite density quantification (QPD). Red indicates low, compatibility, yellow indicates medium compatibility and green indicates high compatibility with POC applications. Gray is employed when data were absent. Abbreviations: *LAMP* loop-mediated isothermal amplification, *PCR* polymerase chain reaction, *CRISPR* Clustered Regularly Interspaced Short Palindromic Repeats

Figure 3 summarizes the average scores assigned to ASSURED criteria, TRL, SID, QPD and LOD for each analytical method reported by at least 3 records in the form of a heatmap. Although not all records included the necessary information to evaluate all the metrics, the heatmap highlights the fact that no single analytical method exhibits consistently high compatibility with all ASSURED criteria. A more detailed breakdown of scores is reported in Table SI4, where immunoassays—which are more frequently reported than the other assays—were further subdivided according to the readout technology.

The capacity to identify species and quantify parasite density are summarized in Table 2.

**Table 2:** Species identification and parasitaemia quantification for the analytical assays

Analytical assay	Species identification	Quantification of parasite density	Reference(s)
Immunoassay	Pf, Pv	No	[66–68]
LAMP	Pf, Pv, Pm, Po, Pk	Yes	[69, 70]
PCR	Pf, Pv, Pm, Po, Pk	Yes	[39, 71]
Optical microscopy	Pf	Yes	[43, 44]
Aptamer binding	Pf, Pv	Yes	[49]
CRISPR	Pf, Pk, Po, Pm, Pv	Yes	[54]
Magneto-optical assay	Pf, Pv	No	[58]
Probe hybridization	Pf, Pv	Yes	[60]

*Pf P. falciparum, Pk P. Knowlesi, Po P. ovale, Pm P. malariae, Pv P. vivax, LAMP* loop-mediated isothermal amplification, *PCR *polymerase chain reaction*, CRISPR *Clustered Regularly Interspaced Short Palindromic Repeat

It is worth noting that the reported data are based only on information present in the published records included in the analysed dataset. Therefore, they are not necessarily representative of the current state-of-the-art of each analytical assay. Additionally, features associated with the diagnostic accuracy of IVD were reported only by a limited number of records (Table SI4). For example, optical microscopy is known for its capacity to identify all species and a skilled microscopist can detect down to 5 parasites/µL, but this is not reflected in Fig. 3 and Table 2. Similarly, PCR is known to have a much lower LOD than that reported in Table SI4 [29].

### Analysis of records by analytical method

#### Immunoassay

Immunoassays exploit the antibody-antigen binding reaction. Different types of immunoassays were identified: enzyme linked immunosorbent assay (ELISA) [30], lateral flow assay [31] or enzyme linked fluorescence assay [32]. Antigens are detected in blood, saliva and urine [33]. The records were stratified according to readout technology as it relates to the compatibility of POC applications in LRS (Table SI4). Most of the ASSURED criteria (*affordable, sensitive* and *specific*) as well as LOD were ranked as medium, while *usability* was ranked as medium/high (depending on the readout technology) and *rapid* and *robust* which were ranked as high. The *equipment free* criterion was ranked medium, while no sufficient information was gathered to give a rank to the *deliverable to end user* criterion. Immunoassays can detect *P. falciparum* and *P. vivax* species but cannot quantify parasite density. The average TRL was 3–4, indicating that these technologies are currently far from commercialization.

#### Loop-mediated isothermal amplification 

LAMP amplifies target DNA sequences at constant temperature and generates turbidity as a byproduct of amplification. Readout can be performed by evaluating turbidity or using dyes which result in visible colour changes with the naked eye. Alternatively, turbidity, absorbance or fluorescence can be measured and quantified with laboratory instruments [34, 35]. *Affordability* was rated as medium since fluorescence readouts require laboratory equipment which require some training to operate. Based on the analysed records, LAMP assays were characterized by medium *sensitivity, rapidity and robustness,* and high *specificity*. The records reported parasite density detection down to 0.01 parasites/µl, thereby LOD was ranked as high. *Equipment free* was ranked as low/medium as simple laboratory equipment is needed. LAMP can quantify parasite density and detect all *Plasmodium* species. An average TRL of 3–4 indicates that most LAMP-based technologies are at a low level of maturity.

#### Polymerase chain reaction

PCR amplifies target DNA sequences using cycles of three different temperatures [36]. Like LAMP, PCR readouts can be performed by fluorescence [37], absorbance [39], or the naked eye [40]. Readout based on changes in resonance frequency (through a quartz crystal microbalance) has also been reported [41]. Although PCR is highly *sensitive* and *specifi*c, a*ffordability* as well as *usability* were rated as medium because of the need for multiple reagents and sophisticated equipment as well as specifically trained personnel. Similarly, although some alternatives to thermal cyclers for amplification of parasite DNA have been proposed [38], in general, *equipment free* was ranked as low. PCR can quantify parasite density and detect all *Plasmodium* species. According to the dataset, LOD was ranked medium. The average technology TRL level is 3–4: this highlights that most of technologies are far from the market but are presented as proof-of-concept design.

#### Optical microscopy

For optical microscopy analysis, a whole blood sample is smeared on a glass slide, dyed with Giemsa stain solution and then observed under a microscope at a magnification of 1000 × to identify the parasite. Readout can be carried out by naked eye [42] or by automated image processing. The parasite can also be detected using specific fluorescent stains that bind to nucleic acids or components of the pathogen [56, 57]. Microscopy can be translated to fit POC by employing portable and custom-made equipment [43, 44]. Because of the need for high magnification optics, we rated optical microscopy medium in terms of *affordability* and low in terms of *equipment, rapidity* and *robustness*. On the other hand, *sensitivity and specificity* were rated high. *Usability* was rated medium since optical microscopy requires training on how to detect the parasite and a high level of expertise. In the analysed records, optical microscopy is employed to detect *P. falciparum* species and can quantify parasite density. LOD was ranked low according to data present in the analysed dataset. The average TRL value of optical microscopy applications analysed in this scoping review was 5–6. However, it should be underlined that some of the records do not regard an entire system for image acquisition analysis but only address part of the diagnostic pipeline, as objective lenses [45] or automated image processing.

#### Aptamer binding

Aptamer binding is a molecular method using single-stranded nucleic acid sequences (i.e. aptamers) to bind and detect proteins. As aptamers are chemically synthesized, they are more stable, easier to produce and less expensive than antibodies [46]. Five papers adopting aptamer binding methodology were included in this scoping review. All the records were published in the 2016–2023 period and therefore quite recent. The readout is performed by analysing an electrochemical signal [47, 48] or an absorbance value [49, 50]. *Affordability*, *sensitivity* and *specificity* were rated as low, while *usability* and *equipment* criteria were rated as medium/low. *Rapidity* was ranked as medium. Aptamer binding can detect *P. falciparum* and *P. vivax* species and quantify parasite density. LOD was ranked as low but was reported in only one record. The average TRL was 1–2, given that all five papers presented a low level of technological maturity.

#### Clustered regularly interspaced short palindromic repeat 

CRISPR-based diagnostics exploits programmable endonucleases (Cas enzymes, Cas 12a in this specific case) that recognize and cleave target nucleic acid sequences guided by guide RNA. In the three records included in this study, whole blood was employed as a biological specimen for testing. The application of this methodology to malaria POC diagnostics is recent since all three papers were published after 2020. The readout can be performed by the naked eye or by fluorescence [51–53]. These systems are characterized by high *sensitivity* and low *specificity.* All the other ASSURED criteria were ranked as medium except for *rapidity and robustness* which ranked high. CRISPR assays can detect all *Plasmodium* species and quantify parasite density. LOD was ranked as high, but was reported in only one record. The average TRL (3–4) highlights how, despite the constraints, researchers working in this field are trying to translate this technology from the lab to the market.

#### Magneto optical assay

Magneto optical assays are employed to detect hemozoin, a nanocrystal which is a byproduct of hemoglobin digestion by malarial parasites [54]. The magnetic properties of hemozoin can be employed to diagnose malaria with different methodologies such as rotating-crystal magneto-optical detection, optical spectroscopy or optical absorption. Readout can be performed by the naked eye [55] or measuring an absorbance signal [56, 57]. Despite there being only three records, the average TRL is high (8–9) since one absorbance-based system is a commercial product [58], while another was tested on 1000 samples [55]. Additionally, a naked eye readout system is presented as a proof-of-concept technology [57]. All ASSURED criteria were ranked medium, except for *rapidity* that ranked high and for *sensitivity* that ranked low. Magneto optical assay can detect *P. falciparum* and *P. vivax* species but cannot quantify parasite density. LOD was ranked as medium–high. Despite there being only three records, the average TRL of these techniques was high (8–9) since one absorbance-based system is a commercial product [56], while another was tested on 1000 samples [55].

#### Probe hybridization

Probe hybridization involves the employment of specific nucleotide probes to bind target parasite DNA or RNA sequences, enabling their detection [58]. Readout can be performed by electrochemical transduction [59], scattering or surface plasmon resonance [60, 61]. All ASSURED criteria were ranked as low, except for *specificity*—ranked medium—and *equipment—*ranked high. This assay can detect *P. falciparum* and *P. vivax* species and quantify parasite density. No data were reported regarding LOD. The TRL of the 3 systems included in this scoping review was 1–2, indicating that the methodology was presented as a proof-of-concept application.

## Discussion

The widespread availability of accurate, accessible, and easy-to-use diagnostic tools for malaria is crucial for reducing the disease’s morbidity and mortality. Understanding the compatibility of current IVDs with POC applications in LRS is essential. This scoping review contributes to the identification of technological deficiencies and to inform strategies to address existing limitations.

The analysis of eligible records revealed that less than 10% of the studies explicitly mentioned the ASSURED criteria despite declaring POC applications as a focus. This observation underscores the need to further disseminate the ASSURED framework among researchers, perhaps through widely consulted resources such as World Malaria Reports or Malaria Technical Strategies. Moreover, involving authors from endemic countries is critical for ensuring that diagnostic solutions are well-aligned with context-specific needs and can be developed locally. However, only 28.8% of the papers had at least one author affiliated to an institution located in malaria endemic countries, indicating a lack of engagement that should be addressed in future research efforts.

Ensuring the compatibility of diagnostic tools with POC applications in LRS also requires engaging with relevant stakeholders, such as health care workers, to understand real-world constraints. In addition to the ASSURED criteria, the WHO’s Target Product Profiles (TPPs) outline the desired characteristics of new health products, particularly for use in LRS. TPPs are intended as a guide to ensure that new medical devices and products are not only scientifically sound but can also be implemented effectively to meet global health needs [62]. Malaria diagnostics TPPs emphasise the importance of species identification (necessary for the choice of the appropriate treatment), the need for high clinical and analytical specificity and sensitivity, and the use of non-invasive sample collection (Table SI5). In the present dataset, species identification was achieved by 85% of methodologies. Non-invasive sampling should also be promoted to minimise disease transmission and facilitate sample collection and storage.

The technologies assessed in this scoping review were analysed in terms of the ASSURED criteria and the level of maturity as measured by the TRL. The findings illustrate that most of the technologies are still at an early stage of product development, with 58.8% classified as proof-of-concept and lacking validation in relevant scenarios (TLR 1–2). Only 7.8% of studies showed a TRL greater than 7, while 34% showed a TRL between 3 and 6.

Emerging technologies such as aptamer binding, CRISPR, magneto optical assay, and probe hybridization remained limited to a small number of studies. This limited dataset indicates a need for further investigation to assess their suitability for POC applications. Based on ASSURED and TRL rankings (Fig. 3), although these technologies may hold promise for the future, they are currently not ready for POC use.

Among the remaining assessed methods, only optical microscopy currently allows identification of the *Plasmodium* species and quantification of parasite density with a single reagent, providing comprehensive diagnostic data. PCR and LAMP can detect and quantify all types of *Plasmodium* species but employing different primers and/or reagents. However, optical microscopy did not meet all ASSURED criteria, particularly in terms of rapidity and the need for equipment (Table SI4 and Fig. 3). Despite the relatively low cost of reagents, the requirement for skilled microscopists and the time-consuming nature of the analysing are significant barriers to implementation in LRS.

To overcome these challenges, we propose enhancing the compatibility of microscopy with the ASSURED criteria by leveraging innovative technologies. Advances in automated image processing and artificial intelligence (AI) algorithms could be integrated to improve microscopy’s compatibility with POC needs, such as standardising *specificity* and *sensitivity* and improving the *rapidity* of the diagnosis. Indeed, AI could mitigate the need for trained personnel in remote settings, while also significantly reducing the time between sample collection and results [43, 63]. From an *equipment-free* perspective, additive manufacturing could enhance access to diagnostic tools even in areas with limited technology supply chains. Local manufacturing could reduce costs, allow on-demand production of spare parts, and facilitate distribution, thereby improving *affordability* and *deliverability.* Moreover, an open-source approach to device design could lead to more effective and safer products while improving user familiarity and maintenance [64, 65].

## Conclusions

In summary, the current landscape of malaria diagnostics shows that no single technology fully meets the ASSURED criteria for LRS. Enhancing the compatibility of microscopy-based methods through AI and local manufacturing could significantly improve their feasibility for POC use. Continued innovation and collaboration, along with harmonized efforts to consider local contexts based on evidence-based requirements developed by on-the-ground experts and users, are needed to provide reliable, accessible, and effective diagnostics in LRS, ultimately contributing to the fight against malaria.

## Supplementary Information

Supplementary material 1.

## Data Availability

The datasets generated and/or analysed during the current study are available from the corresponding author on reasonable request.

## Abbreviations

CRISPR: Clustered Regularly Interspaced Short Palindromic Repeat
ELISA: Enzyme linked immunosorbent assay
GNI: Gross National Income
HRP2: Histidine-rich protein II
IVD(s): In vitro diagnostic(s)
LAMP: Loop-mediated isothermal amplification
LOD: Limit of detection
LRS: Low resource settings
PCR: Polymerase chain reaction
pLDH: *Plasmodium* Lactate dehydrogenase
POC: Point of care
QPD: Quantification of parasite density
RDT(s): Rapid diagnostic test(s)
SI: Supplementary information
SID: Species identification
TRL: Technology readiness level
TTP(s): Target product profile
UN: United Nations
WHO: World Health Organization
