# Supplementary material for: Technologies for the point-of-care diagnosis of malaria: a scoping review
Source: Infect Dis Poverty. 2025 Jun 23;14:54. doi: 10.1186/s40249-025-01329-1 (PMC12183878; doi:10.1186/s40249-025-01329-1)
Supplement: Supplementary file 1 — Supplementary material 1. [file 40249_2025_1329_MOESM1_ESM.docx]

**Eligibility Criteria**

| **Section** | **Inclusion Criteria** | **Exclusion criteria** |
| --- | --- | --- |
| **Concept** | Records regarding malaria | Records regarding other diseases than malaria |
|  | Records regarding diagnosis | Records regarding prevention, treatment or clinical manifestations were excluded |
|  | Records regarding the development of POC IVDs | Records with a lack of focus on POC IVDs |
| **Context** | Research conducted in any country | - |
|  | All socio-cultural environments in which research was performed were included | - |
|  | Research conducted in any type of healthcare or research setting | - |
| **Types of evidence sources** | Primary research articles published in peer-reviewed journals | Records reporting a review of the literature, case studies, opinion papers, letters, conference abstracts and grey literature |
|  | Written in English | Not written in English |
|  | Full text available | Full text not available |
|  | Published between 2003 and 2023 | Published before 2003 and after 2023 |
|  | Already published | Accepted papers or those in publication were excluded |

Table SI1: Inclusion and exclusion criteria.

**Search terms**

Table SI2 reports the database, the query, and the number of articles each database provided, according to the inclusion and exclusion criteria.

| **Database** | **Boolean operator** | **Search term** | **Variations of the search terms** | **Field** | **Papers** |
| --- | --- | --- | --- | --- | --- |
| Pubmed | **OR** | Malaria | “malaria” OR “plasmodium” | [text word] | 585 |
|  | **AND** | Diagnosis | “diagnos*” OR “detect*” | [text word] |  |
|  | **AND** | Point-of-care | “point of care” OR “point-of-care” OR “poc” | [text word] |  |
|  | | | | | |
| Web of Science | **OR** | Malaria | “malaria” OR “plasmodium” | [topic] | 501 |
|  | **AND** | Diagnosis | “diagnos*” OR “detect*” | [topic] |  |
|  | **AND** | Point-of-care | “point of care” OR “point-of-care” OR “poc” | [topic] |  |
|  | | | | | |
| Scopus | **OR** | Malaria | “malaria” OR “plasmodium” | [title-abs-key] | 863 |
|  | **AND** | Diagnosis | “diagnos*” OR “detect*” | [title-abs-key] |  |
|  | **AND** | Point-of-care | “point of care” OR “point-of-care” OR “poc” | [title-abs-key] |  |

Table SI2: Databases queries and number of papers. In all databases, queries were not case sensitive. Topic in the “field” column refers to title, abstract and keyword.

The eligibility criteria were applied during the query in Scopus, Web of Science and Pubmed, except for the language eligibility criterion that was applied after data extraction. The Pubmed database does not allow research on author keywords. To avoid skipping articles that might be eligible for the study, the research was carried out on the *'text word'* field, which includes the *'title'*, *'abstract'* and ‘*author keywords’* fields.

**Data extraction**

Data were extracted from each database in a .csv file and imported into an Excel template containing the following information for each paper: authors, title, source title, document type, author keywords publication year, DOI, DOI link, and Pubmed ID (where present). The Pubmed ID was used to eliminate duplicates. Where absent, duplicate elimination was performed analysing the title and author list.

**Data Analysis**

| **Biomarker/analyte** | **Analytical method** | **Read out technology** | **Number of papers (% of all papers)** |
| --- | --- | --- | --- |
| Antigen | Immunoassay | Absorbance | 3 |
|  |  | Electrochemical signal | 9 |
|  |  | Fluorescence | 10 |
|  |  | Naked eye | 17 |
|  |  | Other signals | 10 |
|  |  | **Total (% of all papers)** | **49 (42%)** |
|  | Aptamer binding | Absorbance | 2 |
|  |  | Electrochemical signal | 3 |
|  |  | **Total** | **5 (4%)** |
|  | Others | Fluorescence | 2 |
|  |  | **Total** | **2 (2%)** |
| DNA | Clustered Regularly Interspaced Short Palindromic Repeat | Naked eye / Fluorescence | 3 |
|  |  | **Total** | **3 (3%)** |
|  | Loop-mediated isothermal amplification | Electrochemical signal | 1 |
|  |  | Fluorescence | 8 |
|  |  | Naked eye | 16 |
|  |  | Other signals | 2 |
|  |  | **Total** | **27 (23%)** |
|  | Polymerase Chain Reaction | Absorbance | 1 |
|  |  | Fluorescence | 4 |
|  |  | Naked eye | 2 |
|  |  | Other signals | 1 |
|  |  | **Total** | **8 (7%)** |
|  | Probe hybridization | Electrochemical signal | 1 |
|  |  | Other signals | 2 |
|  |  | **Total** | **3 (3%)** |
| Enzyme (TopoI) | Other - Rolling-Circle-Enhance-Enzyme-Activity-Detection | Fluorescence | 2 |
|  |  | **Total** | **2 (2%)** |
| Hemozoin | Magneto optical assay | Absorbance | 2 |
|  |  | Naked eye | 1 |
|  |  | **Total** | **3 (3%)** |
|  | Other | Absorbance | 2 |
|  |  | Other signals | 2 |
|  |  | **Total** | **4 (3%)** |
| Parasite | Optical microscopy | Florescence | 3 |
|  |  | Naked eye | 3 |
|  |  | **Total** | **6 (5%)** |
|  | Other | Absorbance | 3 |
|  |  | Other signal | 1 |
|  |  | **Total** | **4 (3%)** |

Table SI 3: Number of papers for the different biomarkers, analytical methods and read out technologies.

| **Assay (#papers)** | | **A** | **S (#reported)** | **S (#reported)** | **U** | **R** | **E** | **D** | **TRL^a^** | **LOD (#reported)** | **SID** | **QPD** |
| --- | --- | --- | --- | --- | --- | --- | --- | --- | --- | --- | --- | --- |
| Immunoassay^b^ (49) | Naked eye (17) | Medium | Medium (3) | Medium (3) | Medium/High | High | High | N.A. | 3-4 | High (2) | Pf, Pv | No |
|  | Absorbance (3) | Low | N.A. | N. A. | Low | Medium | Low | N.A. | 1-2 | N.A. | Pf, Pv | No |
|  | Fluorescenze (10) | Medium | High (1) | High (1) | Low | Medium | Low | N.A. | 3-4 | Medium (3) | Pf, Pv | No |
|  |  |  |  |  |  |  |  |  |  |  | Pf, Pv | No |
|  | Electrochemical and others (19) | Low | Low (1) | High (1) | Medium | High | Medium | N.A. | 1-2 | Low (2) | Pf, Pv | No |
| LAMP (27) | | Medium | Medium (12) | High (13) | Medium | Medium | Low/ Medium | N.A. | 3-4 | High (11) | Pf, Pv, Pm, Po, Pk | Yes |
| PCR (8) | | Medium | High (4) | High (4) | Medium | Low | Low | N.A. | 3-4 | Medium (4) | Pf, Pv, Pm, Po, Pk | Yes |
| Optical microscopy (6) | | Medium | High (1) | High (1) | Medium | Low | Low | N.A. | 5-6 | Low (1) | Pf | Yes |
| Aptamer binding (5) | | Low | Low (1) | Low (1) | Low/medium | Medium | Low/medium | N.A. | 1-2 | Low (1) | Pf, Pv | Yes |
| CRISPR (3) | | Medium | High (1) | Low (1) | Medium | High | Medium | N.A. | 3-4 | High (2) | Pf, Pv, Pm, Po, Pk | Yes |
| Magneto optical assay (3) | | Medium | Low (2) | Medium (2) | Medium | High | Medium | N.A. | 5-6 | Medium/High (2) | Pf, Pv | Yes |
| Probe hybridization (3) | | Low | High (1) | Medium (1) | Low | High | Low | N.A. | 1-2 | N.A. | Pf, Pv | Yes |

^a^: TRL ranges

^b^: Average rankings for all immunoassays. Differences between different types of readout are provided only for this type of assay.

Table SI4: ASSURED criteria ranked low, medium, or high for POC applications, TRL and LOD (also ranked). Species identification (SID) and parasitaemia quantification (QPD) for each analytical assay. Pf: P. falciparum, Pk: P. Knowlesi, Po: P. ovale, Pm: P. malariae, Pv: P. vivax. Numbers in brackets refer to the number of records reporting sufficient information to give a rank to the criteria.

**Target product profile in WHO database**

Table SI5 reports the characteristics of active target product profile regarding malaria diagnosis.

| **Indication** | **Year** | **Intended use** | **Target population** | **Sample type and volume** | **Use settings** | **Minimal Performance** | **Optimal performance** |
| --- | --- | --- | --- | --- | --- | --- | --- |
| TPP PvB1: Point-of-care diagnosis of sub-clinical P. vivax infection | 2017 | For parasitological confirmation of all infections of P. vivax malaria (symptomatic and asymptomatic) | All individuals susceptible to suffer from P. vivax infection in endemic settings | Minimal sample type: Capillary blood  Minimal sample volume: <= 100 μL | Community health facilities, health posts, health centers. Target users (Minimal): Community and facility-based health workers | Analytical Sensitivity: 20 p/µL  Diagnostic sensitivity: >95%  Diagnostic specificity: >95% | Analytical Sensitivity: 1 p/µL  Diagnostic sensitivity: >99%  Diagnostic specificity: >99% |
| TPP PvB2: Population screening for Plasmodium vivax infection surveillance | 2017 | For indication of present or recent P. vivax infection for epidemiological surveys and surveillance activities | All individuals in an endemic setting | Minimal sample type: Capillary blood  Minimal sample volume: <= 200 μL | Implementation level: District hospitals and reference. laboratories. Target users (Minimal): Laboratory technicians | Analytical Sensitivity: 0.1 p/µL  Diagnostic sensitivity: >95%  Diagnostic specificity: >95% | Analytical Sensitivity: 0.01 p/µL  Diagnostic sensitivity: >99%  Diagnostic specificity: >99% |
| TPP PvA: Diagnosis of P. vivax malaria acute infection | 2017 | For parasitological confirmation of symptomatic suspected cases of P. vivax malaria | All individuals suspected to suffer from clinical P. vivax infection | Minimal sample type: Capillary blood  Minimal sample volume: <= 100 μL | Community health facilities, health posts, health centers. Target users (Minimal): Community and facility based health workers | Analytical Sensitivity: 25 p/µL  Diagnostic sensitivity: >95%  Diagnostic specificity: >95% | Analytical Sensitivity: 5 p/µL  Diagnostic sensitivity: >99%  Diagnostic specificity: >99% |
| Radically new and improved approaches to traditional immune and molecular assay methods  Biochemical amplification or analysis of non-invasive samples such as urine, saliva, sweat or other excreted fluids | 2015 | - | - | - | - | - | - |
| Malaria | 2014 | Minimal requirement: Active infection detection (ID) interventions aimed at low-density and subclinical infection detection.  Optimal: Active infection detection (ID) interventions aimed at low-density and subclinical infection detection, passive case detection for clinical diagnosis and management, and epidemiological surveys | Minimal requirement: Individuals with Plasmodium falciparum (P. falciparum; Pf) infection, whether or not they have symptoms of infection.  Optimal: Individuals with any malaria species infection, whether or not they have symptoms of infection | Minimal Sample Type: Peripheral whole blood from finger stick (heel prick for infants)  Minimal Sample Volume: 1‒50 µl.  Optimal Sample Type: Less invasive sample types that do not include finger stick (e.g., saliva, buccal).  Optimal Sample Volume: 1-25 µl | Minimal requirement: Lowest level user: The test will be performed by community health workers, trained lay persons, and community volunteers.  Optimal: Lowest level user: All adults.  Lowest infrastructure level (Minimal & optimal): The test will be performed under zero-infrastructure conditions including community health centers, households, and outdoor conditions. | Pf only.  Analytical Sensitivity: 10 p/µL  For HRP2, the requirement is 12 ng/ml.  Diagnostic sensitivity: >97%  Diagnostic specificity: >90% | Pf /Pv/Ppan  Analytical Sensitivity: 5 p/µL  For HRP2, the requirement is 6 ng/ml.  Diagnostic sensitivity: >99%  Diagnostic specificity: >99% |
| Malaria | 2014 | Minimal requirement: Active infection detection (ID) interventions aimed at low-density and subclinical infection detection.  Optimistic specification: Active ID interventions aimed at low density and subclinical infection detection; passive case detection for clinical diagnosis and management; epidemiological surveys. | Minimal requirement: Individuals with Plasmodium falciparum (P. falciparum; Pf) infection, whether or not they have symptoms of infection  Optimistic specification: Individuals with any malaria species infection, whether or not they have symptoms of infection | Minimal requirement: Peripheral whole blood from finger stick (heel prick for infants), 1‒50 µl  Optimistic specification: Less invasive sample types that do not include finger stick (e.g., saliva, buccal), 1‒25 µl. | Lowest infrastructure level/ lowest level user: Performed under zero infrastructure conditions including community health centers, households and outdoor conditions by community health workers, trained lay persons and community volunteers; in optimistic case, all adults | Analytical Sensitivity: 10 p/µL  For HRP2, the requirement is 12 ng/ml  Diagnostic sensitivity: >97%  Diagnostic specificity: >90% | Analytical Sensitivity: 5 p/µL  For HRP2, the requirement is 6 ng/ml  Diagnostic sensitivity: >99%  Diagnostic specificity: >99% |
| • Accurate, sensitive POC RDTs(Point of Care Rapid Diagnostic Tests), specifically asymptomatic carrier.  • RDT for Plasmodium falciparum and/or vivax with 2 logs better sensitivity and accuracy compared to current RDT product. | 2013 | - | - | - | - | - | - |

Table SI5: TTPs present in the database with specifically indications about year, intended use, target population, sample type and volume, use settings, minimal performance and optimal performance.
